# Structural and Functional Profiling of Water-Extracted Polypeptides from *Periplaneta americana*: A Multifunctional Cosmetic Bioactive Agent with Antioxidative and Anti-Inflammatory Properties

**DOI:** 10.3390/molecules30142901

**Published:** 2025-07-09

**Authors:** Xinyu Sun, Zhengyang Zhang, Jingyao Qu, Deyun Yao, Zeyuan Sun, Jingyi Zhou, Jiayuan Xie, Mingyang Zhou, Xiaodeng Yang, Ling Wang

**Affiliations:** School of Chemistry and Chemical Engineering, Qilu University of Technology (Shandong Academy of Sciences), Jinan 250353, China; sxx1615020756@163.com (X.S.); zqezzy@163.com (Z.Z.); 13371585685@163.com (J.Q.); 13678677663@163.com (D.Y.); 19560653855@163.com (Z.S.); z1227318380@163.com (J.Z.); xie908557198@163.com (J.X.); myzhou@qlu.edu.cn (M.Z.); yangxiaodeng@qlu.edu.cn (X.Y.)

**Keywords:** *Periplaneta americana*, bioactive ingredients, skincare, anti-aging, anti-allergic activity

## Abstract

Low-molecular-weight polypeptides (<3 kDa) were prepared from Periplaneta americana via enzymatic hydrolysis and ultrafiltration, yielding 3.53 ± 0.01 mg/g of peptide-rich extract. The extract was primarily composed of peptides, proteins, polysaccharides, phenolics, and flavonoids. HPLC-MS analysis identified 1402 peptide sequences, 80.51% of which were below 1000 Da, predominantly consisting of tri-, tetra-, and octapeptides. Monosaccharide profiling detected D-(+)-galactose, and quantitative assays determined the contents of total phenolics (12.28 mg/g), flavonoids (15.50 mg/g), proteins (85.84 mg/g), and total sugars (17.62 mg/g). The biological activities of the extract were systematically evaluated. The peptide fraction inhibited hyaluronidase activity by 58% at 5 mg/mL, suggesting protection of extracellular matrix integrity. In HaCaT keratinocytes, it promoted cell proliferation by 62.6%, accelerated scratch wound closure by 54%, upregulated Wnt-10b and β-catenin expression, and reduced intracellular ROS levels under oxidative stress. In LPS-stimulated RAW 264.7 macrophages, the extract decreased TNF-α, IL-6, and IL-1β production by 30%, 25%, and 28%, respectively, reduced MDA levels by 35.2%, and enhanced CAT and SOD activities by 12.3% and 60.3%. In vivo, complete closure of full-thickness skin wounds in mice was achieved by day 14. Safety evaluations using the chick chorioallantoic membrane assay and human patch tests confirmed the extract to be non-irritating and non-toxic. These findings highlight *Periplaneta americana* extract as a promising multifunctional bioactive ingredient for cosmetic and dermatological applications. Further studies on its active components, mechanisms of action, and clinical efficacy are warranted to support its development in skin health and aesthetic medicine.

## 1. Introduction

As consumer demand for the natural properties, safety, and efficacy of cosmetic ingredients continues to rise, the use of natural bioactive compounds in cosmetics has gained significant attention. Natural extracts are particularly valued for their roles in anti-skin aging, antioxidant activities, and anti-inflammatory effects. Their limited side effects and superior biocompatibility make them essential components in contemporary skincare formulations. Consequently, researching the biological activity of natural extracts, understanding their effects and mechanisms for skin protection, and providing theoretical support for their application within the skincare industry has become a focal point of study.

*Periplaneta americana*, commonly known as the American cockroach, is an insect found throughout the world. It is rich in bioactive compounds and possesses notable nutritional and medicinal properties [1,2]. Research conducted by Bai identified nitrogen-containing phenolic compounds in extracts of *Periplaneta americana*, which demonstrate inhibitory effects against triple-negative breast cancer. Their findings indicate that these compounds may have therapeutic potential for cancer treatment [3]. Additionally, Guo explored the use of a Schiff base hydrogel dressing infused with *Periplaneta americana* extracts for diabetic wound healing. The results revealed that this hydrogel significantly enhanced wound healing, suggesting a promising therapeutic application of *Periplaneta americana* extracts in wound care [4]. Ren identified immunosuppressive metabolites derived from the insect-associated endophyte fungus *Aspergillus taichungensis* in *Periplaneta americana*, underscoring their potential for immune modulation [5]. The extraction methodology significantly affects the compositional profile and bioactivity of *Periplaneta americana* extracts (PAE). Aqueous extracts predominantly contain hydrophilic compounds such as polysaccharides and proteins, while enzymatic hydrolysis enhances the release of higher molecular weight bioactive peptides [6]. Comparative studies reveal that enzymatic extraction improves antioxidant and anti-inflammatory properties compared to traditional solvent-based methods. For example, Liao et al. discovered four overlapping peptide fractions (PaPPc1–PaPPc4) in aqueous PAE, with PaPPc2 and PaPPc3 exhibiting notable wound-healing capabilities [6]. Likewise, Nguyen et al. found that PAE reduced oxidative tissue damage and inhibited inflammatory pathways through NF-κB inhibition and modulation of the MAPK pathway [7]. Ethanol-based extraction produces distinct phytochemical profiles. Luo et al. isolated four isocoumarin derivatives from 70% ethanolic extracts, while Pang et al. purified two glycoproteins (PAGP-1 and PAGP-2) from 95% ethanol extracts, both showcasing amino acid diversity and dose-dependent bioactivity [8,9]. Notably, 75% ethanolic PAE lessened neuronal damage in Parkinson’s disease models by suppressing AKT-mediated endoplasmic reticulum stress [10]. Extraction efficiency and biological efficacy are influenced by the source of the specimen and the parameters involved in processing. Zhu et al. demonstrated that ethanol-extracted PAE achieved superior wound closure rates compared to polysaccharide or protein fractions, which was linked to accelerated HaCaT cell migration through the activation of the JAK/STAT3 pathway [11,12]. These findings highlight the importance of optimizing extraction methods to enhance the therapeutic potential. A systematic comparison of extraction techniques—covering solvent selection, enzymatic treatment, and variability in the source—will facilitate the rational development of PAE-based skincare formulations aimed at addressing oxidative stress, inflammation, and tissue regeneration. Additionally, Bai et al. identified eight novel compounds from the 70% ethanol extract of *Periplaneta americana*, including pyrrole-2-carboxaldehyde derivatives (periplanpyrroles A–D), spirooxindoles (perispirooxindoles A–B), and phenolic analogs (periplanetols G–H), alongside eight known compounds. The structural elucidation of these compounds was achieved through comprehensive spectroscopic (NMR, MS) and computational analyses. Notably, periplanetol G demonstrated significant anti-metastatic activity in triple-negative breast cancer (TNBC) models, inhibiting BT549 and MDA-MB-231 cell migration while downregulating EMT markers (vimentin and N-cadherin) in a dose-dependent manner [3]. In a complementary study, Zhao et al. demonstrated that the extract of *Periplaneta americana* accelerated the healing of mucosal wounds on the hard palate by enhancing keratinocyte migration and collagen synthesis through the PI3K/AKT signaling pathway, as confirmed by RNA sequencing and pharmacological inhibition (LY294002) [13]. Further advancements in drug delivery were achieved by Wu et al., who developed a thermosensitive in situ gel (PA/CCMTS-P) through Schiff base crosslinking. This formulation exhibited a phase transition at 32.9 °C and protected the intestinal mucosal barrier in ulcerative colitis models by modulating NF-κB/NLRP3 signaling pathways [14]. Expanding beyond biomedical applications, Chen et al. explored the potential of chitosan films derived from *Periplaneta americana* (PaCSF) for food packaging. Their findings revealed that PaCSF exhibited superior UV resistance, antioxidant properties, and antibacterial efficacy against *Serratia marcescens* and *Escherichia coli* when compared to shrimp-derived chitosan films, highlighting its promise as a sustainable biomaterial [15]. Furthermore, Liao et al. successfully isolated extracellular vesicles (PA-ELNs) from *Periplaneta americana* using differential centrifugation. PA-ELNs significantly enhanced fibroblast proliferation and aided diabetic wound healing, achieving nearly complete wound closure (97%) by day 14 while simultaneously reducing pro-inflammatory cytokines (IL-6, TNF-α) in murine models [16]. These results align with the escalating demand for natural bioactive ingredients in cosmetics, driven by a global market valued at 800 billion USD in 2023, with an annual growth rate of 7%. By addressing both intrinsic and extrinsic factors of skin aging, extracts from *Periplaneta americana* offer promising alternatives for improving skin health and appearance [17].

The increasing consumer demand for multifunctional cosmetic ingredients has generated interest in naturally derived bioactives that possess antioxidative, anti-inflammatory, and wound-healing properties. In particular, peptides—especially those characterized by low molecular weight—have garnered significant attention due to their remarkable skin-penetration capabilities and diverse biological activities, which include free-radical scavenging, enzyme inhibition, and the modulation of cellular signaling pathways. Insects, which have long been utilized in traditional medicine, present an underexplored source of such peptides. Extracts from *Periplaneta americana* (the American cockroach) have historically been used for tissue despite the promising potential of *Periplaneta americana* extracts for cosmetic applications, there remain several gaps in the existing literature. Most studies concentrate on a single extraction method and basic compositional analysis without systematically comparing various extraction techniques and their effects on bioactive compounds. Moreover, while many studies assess the biological activities of these extracts, few explore their stability, formulation development, and actual application in cosmetic products. These gaps underscore the need for more comprehensive research that not only evaluates the bioactivity of the extracts but also considers the influence of extraction methods and raw material sources on the overall efficacy of the extracts in cosmetic formulations. Although direct stability testing (e.g., thermal or pH conditions) was not conducted, the peptides demonstrated sustained antioxidant, anti-inflammatory, and wound-healing activities under various biological models. These results indirectly suggest good functional stability, which should be further validated through comprehensive physicochemical and formulation studies.

This study focuses on optimizing the processes to enhance the bioactivity and skincare applications of extracts from *Periplaneta americana*. We report on the successful isolation of peptides under three kDa from *Periplaneta americana* through enzymatic hydrolysis and ultrafiltration, followed by a thorough chemical and biological evaluation. High-performance liquid chromatography-mass spectrometry (HPLC-MS) was utilized to delineate the peptide sequence distribution, while monosaccharide profiling was conducted to examine the glycoconjugate matrix. We quantified total phenolics, flavonoids, proteins, and sugars to evaluate the extract’s antioxidant potential. Functional assays were performed, including hyaluronidase inhibition to assess extracellular matrix protection, as well as tests for HaCaT keratinocyte proliferation, scratch-wound closure, Wnt/β-catenin activation, and reactive oxygen species (ROS) scavenging to determine skin-regenerative and antioxidative effects. Additionally, RAW 264.7 macrophage assays were carried out to evaluate anti-inflammatory and antioxidant enzyme activities. We further confirmed the in vivo wound-healing efficacy in a murine model, assessed biosafety using the chick chorioallantoic membrane (CAM) assay, and verified dermal compatibility through human patch testing. This comprehensive approach lays a solid foundation for advancing *Periplaneta americana*–derived peptides as next-generation cosmetic bioactives.

## 2. Results

**Figure 1:**
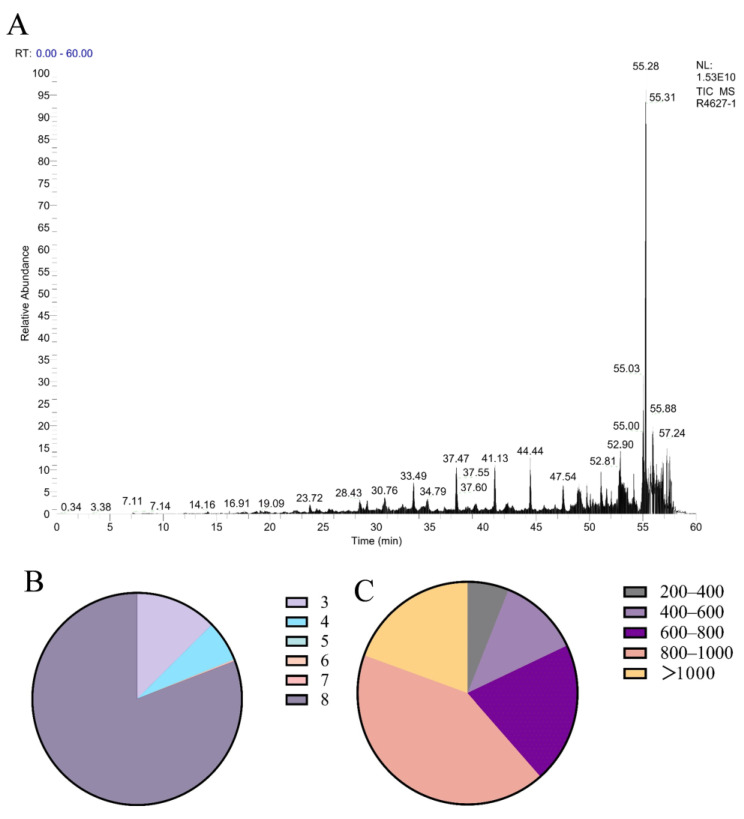
Characterization of *Periplaneta americana* peptides. (**A**) Molecular weight distribution of peptides isolated from PAE. (**B**) Classification and distribution of different peptide types within PAP based on amino acid composition and sequence length. (**C**) Total ion chromatogram (TIC) of PAP obtained by HPLC-MS analysis.

**Figure 2:**
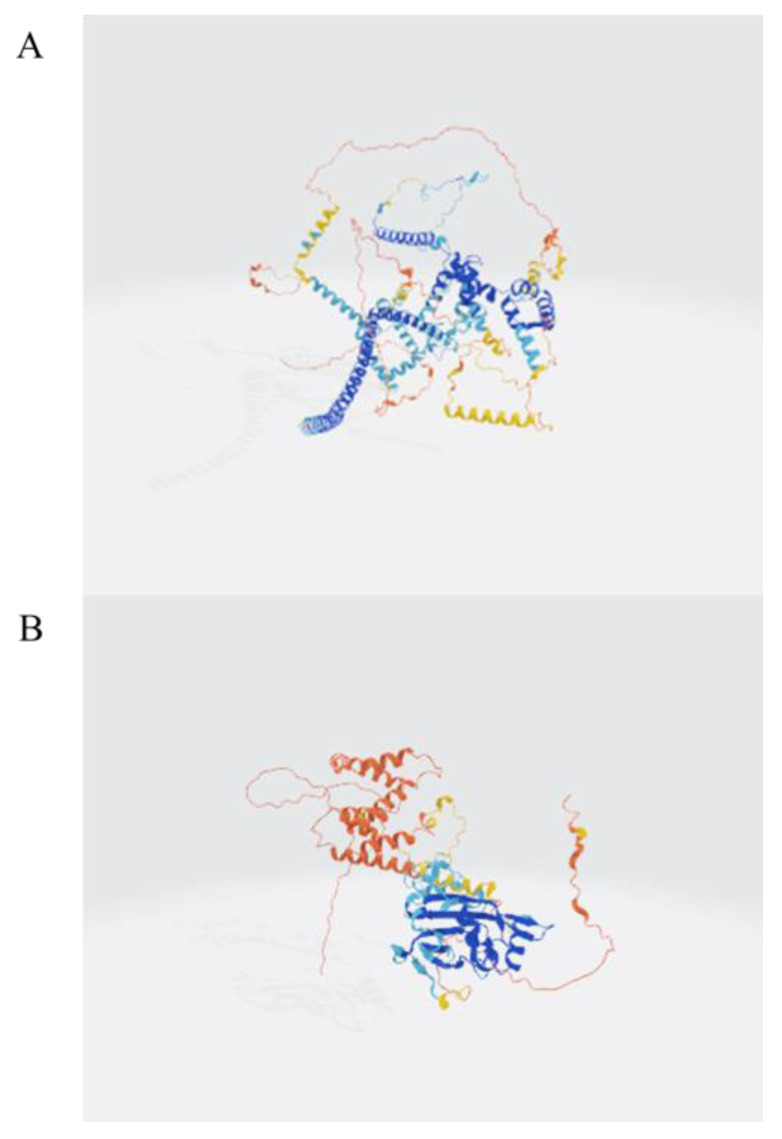
Predicted tertiary structures of representative parent proteins. (**A**) Predicted tertiary structure of UniProt ID: A0A6L2PY33 using AlphaFold. (**B**) Predicted tertiary structure of UniProt ID: A0A6L2Q417 using AlphaFold. These visualizations support the correlation between peptide location and potential bioactive domains within the protein structure. Color coding reflects the per-residue confidence score (pLDDT) predicted by AlphaFold: Very high confidence (pLDDT > 90), dark blue; high confidence (70 < pLDDT ≤ 90), blue; low confidence (50 < pLDDT ≤ 70), orange; very low confidence (pLDDT ≤ 50), red. Residues with low pLDDT scores may correspond to intrinsically disordered regions or flexible loops.

Polypeptides with a molecular weight below 3000 Da were isolated from *Periplaneta americana* through ultrafiltration, to quantify the peptide yield, a BCA protein assay was conducted using the enzymatically hydrolyzed extract. The peptide concentration in the extract was measured to be 3.53 ± 0.01 μg/mL. Given that the extraction was performed using 1 mg/mL of PA powder, this value corresponds to a peptide yield of 3.53 ± 0.01 mg/g of dry powder. The BCA assay showed an increase in total protein concentration from 16.59 mg/mL in the crude aqueous extract (without enzyme treatment) to 26.85 mg/mL after enzymatic hydrolysis. This increase is attributed to the exposure of more peptide bonds and chromogenic amino acid residues, such as tryptophan and tyrosine, following proteolytic cleavage. These structural changes enhance the BCA reagent’s ability to detect protein content. Therefore, the elevated absorbance is considered an indirect indicator of successful enzymatic hydrolysis and the release of peptide-rich fractions. This metric reflects the extraction efficiency and provides a useful reference for comparing with other peptide-based hydrolysates. The analysis conducted using liquid chromatography-mass spectrometry (LC-MS) produced a total ion current (TIC) chromatogram, illustrated in Figure 1A. A total of 1504 MS/MS spectra were obtained, leading to the successful identification of 1402 peptide sequences. The molecular weight distribution of the identified peptides is displayed in Figure 1B, revealing that the majority of peptides were concentrated in the 800–1000 Da range, with 80.51% of peptides falling below 1000 Da. As shown in Figure 1C, tripeptides, tetrapeptides, and octapeptides were the most prevalent, comprising 12.95%, 6.35%, and 82.66% of the identified peptides, respectively. To further explore the structural origin of identified peptides, the parent proteins were analyzed using AlphaFold-based 3D structure prediction. A representative predicted structure of the protein (UniProt ID: A0A6L2PY33 and A0A6L2Q417) is shown in Figure 2, where the cleavage sites of several identified peptides are annotated in red. These peptides are mainly located in surface-exposed loops or flexible domains, suggesting a structural basis for their potential biological activities. Full predicted structures of additional source proteins are included in Figure 2 and Supplementary Materials. Although a total of 1402 peptide sequences were identified by HPLC-MS, including dipeptides to octapeptides, their tertiary structures were not characterized in the current study. Therefore, we recognize the need for future work to focus on structural elucidation through NMR or X-ray crystallography to better understand their biological functions.

**Figure 3:**
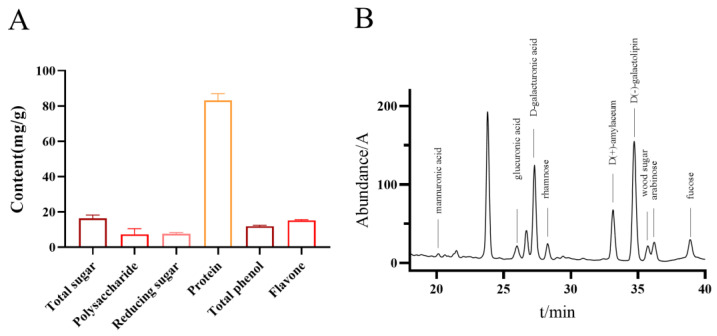
Chemical composition analysis of *Periplaneta americana* extract. (**A**) Quantification of total sugars, polysaccharides, reducing sugars, proteins, total phenolics, and flavonoids in PAE. (**B**) Identification of monosaccharide components in PAE polysaccharide hydrolysates by HPLC analysis.

The total contents of sugar, protein, polyphenols, and flavonoids, as determined using Fehling’s reagent, Bradford, Folin-Ciocalteu, and flavonoid detection kits, were 17.62 mg/g, 85.84 mg/g, 12.28 mg/g, and 15.50 mg/g, respectively (Figure 3A). The monosaccharide composition of the water-extracted polypeptide fraction from *Periplaneta americana* was analyzed using HPLC. A total of ten monosaccharides were identified, with D-(+)-galactose (95.922 mg/L), D-glucose (63.572 mg/L), and D-(+)-mannose (53.152 mg/L) being the most prevalent. Other sugars detected included D-glucuronic acid (13.855 mg/L), D-xylose (15.704 mg/L), arabinose (12.126 mg/L), and rhamnose (23.769 mg/L). Additionally, minor components such as L-guluronic acid (2.004 mg/L), a D-glucuronic acid isomer, and sorbose were found at lower concentrations. The total monosaccharide content was quantified at 294.74 mg/L. These findings suggest that the peptide extract is rich in a variety of neutral and acidic monosaccharides, which may play synergistic or structural roles contributing to its observed bioactivities (Figure 3B).

**Figure 4:**
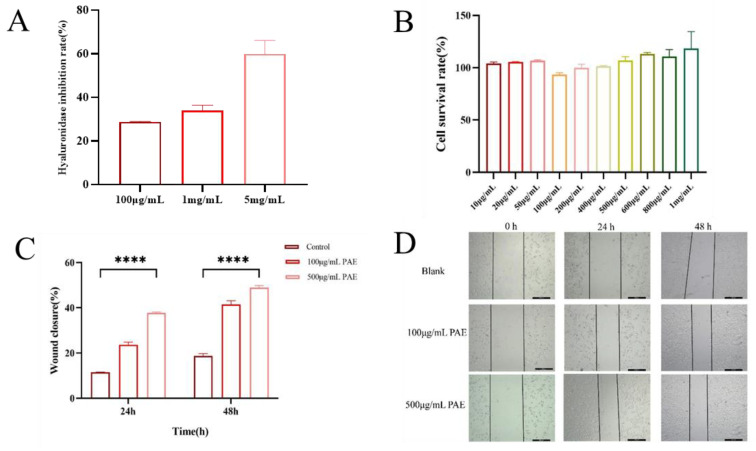
In vitro bioactivity and biocompatibility evaluation of *Periplaneta americana* extract. (**A**) Hyaluronidase inhibitory activity of PAE at concentrations of 100 μg/mL, 1 mg/mL, and 5 mg/mL. (**B**) In vitro biocompatibility and proliferative bioactivity of PAE at different concentrations. (**C**) Scratch wound closure of HaCaT keratinocytes at 0, 24, and 48 h after treatment with PAE. (**D**) Relative proliferation rates of HaCaT cells cultured in PAE solution on days 1 and 2. Statistical significance: **** *p* < 0.0001.

**Figure 5:**
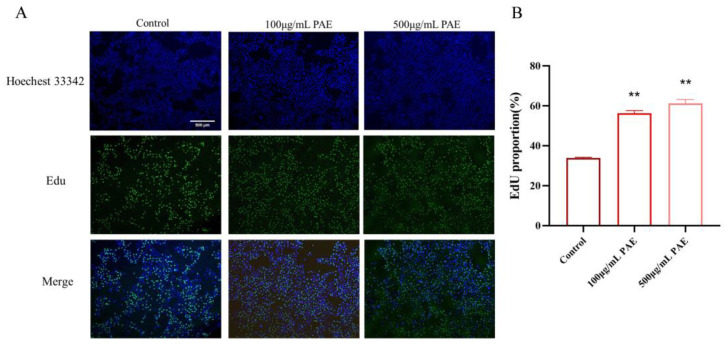
PAE promotes proliferation of HaCaT keratinocytes. (**A**) EdU staining of HaCaT cells treated with PAE at different concentrations. Green fluorescence indicates EdU-positive proliferating cells; blue fluorescence indicates nuclei stained with DAPI. (**B**) Proliferation rate of HaCaT cells quantified by EdU assay. ** *p* < 0.01.

The hyaluronidase inhibitory activity of an extract derived from the American cockroach (*Periplaneta americana*) was assessed using a quantitative assay that detects β-N-acetylglucosamine, a byproduct of sodium hyaluronate degradation. The extract exhibited a dose-dependent inhibition of hyaluronidase, with inhibition rates of 28%, 35%, and 58% at varying concentrations. These findings indicate that the extract has notable hyaluronidase inhibitory potential, especially at higher concentrations. The significant inhibition of hyaluronidase activity is evidenced by a reduction in β-N-acetylglucosamine formation compared to control reactions (Figure 4A). The biocompatibility of PA extracts was evaluated using HaCaT human immortalized epidermal cells. The CCK-8 assay was employed to examine the effects of PAE on cell viability, cytotoxicity, and proliferation. Compared to the blank control (DMEM), the PAAP groups demonstrated enhanced cell viability, indicating that the PA extract significantly promoted cell proliferation (see Figure 4B). The results regarding cytotoxicity and cell proliferation further confirmed the excellent biocompatibility of PA extracts. The scratch healing rate is illustrated in Figure 4D. Notably, a significantly greater degree of cell migration was observed at both 24 h and 48 h in the PAE groups compared to the control group, suggesting that PA extract could facilitate wound healing (refer to Figure 4C,D). Additionally, the number of EDU-positive cells increased significantly in the PAE groups. Fluorescent imaging revealed more green fluorescence (EDU) in the PAE-treated cells, indicating active DNA synthesis. The percentage of EDU-positive cells was significantly higher in the PAE-treated group (*p* < 0.01), further confirming that PAE stimulated cell proliferation (Figure 5).

**Figure 6:**
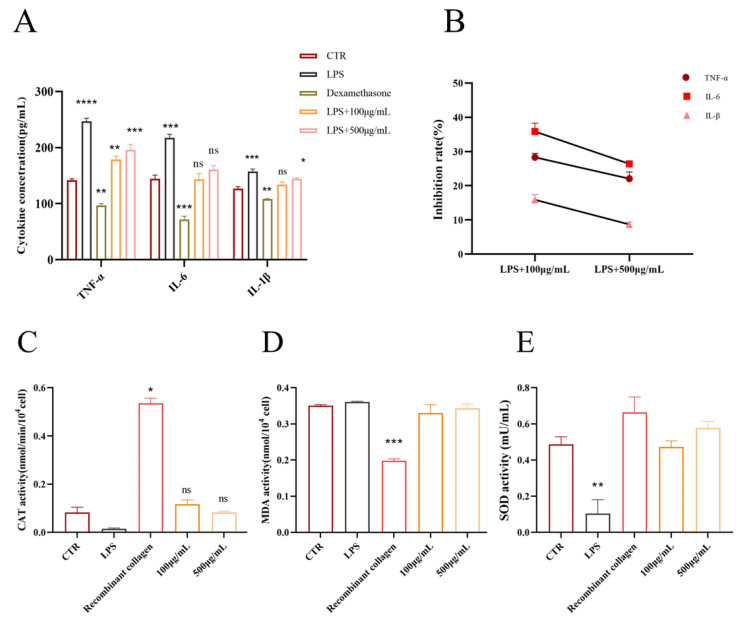
PAE alleviates inflammation and oxidative stress in LPS-stimulated RAW264.7 cells. (**A**) Inflammatory cytokine levels (TNF-α, IL-6, IL-1β) under PAE treatment. (**B**) Reduction in proinflammatory cytokines in LPS-induced cells. (**C**–**E**) Effects of PAE on CAT activity, MDA content, and SOD activity, respectively. * *p* < 0.05, ** *p* < 0.01, *** *p* < 0.001,**** *p* < 0.0001 ns > 0.05, n = 6.

The PAE significantly influenced the secretion of TNF-α, IL-6, and IL-1β from RAW264.7 (see Figure 6A). In RAW246.7 cells treated with 100 μg/mL and 500 μg/mL of PAE, there was a marked reduction in the secretion of TNF-α, IL-6, and IL-1β (*p* < 0.05). Conversely, LPS-stimulated cells (1 μg/mL, *p* < 0.01) exhibited increased secretion of these cytokines, though the treatment effectively reduced TNF-α levels in LPS-induced RAW264.7 cells. Compared to the control group, the PAE-treated group demonstrated significant inhibition at various concentrations, achieving an inhibition rate as high as 30%. For IL-6 levels, PAE treatment notably suppressed secretion in LPS-induced RAW264.7 cells, showcasing a dose-dependent decrease (*p* < 0.001). Similarly, PAE treatment led to a significant reduction in IL-1β secretion levels in LPS-induced RAW264.7 cells, with the highest concentration yielding inhibition rates of 30% for TNF-α, 25% for IL-6, and 28% for IL-1β. These findings indicate that PAE effectively reduces the release of inflammatory factors from RAW246.7 cells (see Figure 6B).

LPS treatment resulted in a significant decrease in catalase (CAT) activity, indicating an increase in oxidative stress. However, treatment with both 100 μg/mL and 500 μg/mL concentrations of PAE significantly restored CAT activity, with the 100 μg/mL group showing recovery comparable to that of the positive control, recombinant collagen. This finding suggests that PAE possesses antioxidant properties. Furthermore, LPS treatment led to a marked increase in malondialdehyde (MDA) levels, a known marker of lipid peroxidation. Both concentrations of PAE effectively reduced MDA levels, with the 500 μg/mL group exhibiting a reduction similar to that of the positive control, indicating that the extract can mitigate oxidative damage. The treatment with LPS led to a notable reduction in superoxide dismutase (SOD) activity, reflecting oxidative stress-induced damage. However, the administration of both 100 μg/mL and 500 μg/mL of PAE significantly restored SOD activity. This indicates that PAE enhances cellular antioxidant capacity and may help mitigate oxidative stress. Collectively, these results indicate that PAE has notable antioxidant effects, is capable of reducing oxidative stress and damage induced by LPS, and is effectively comparable to recombinant collagen. (Figure 6C–E).

**Figure 7:**
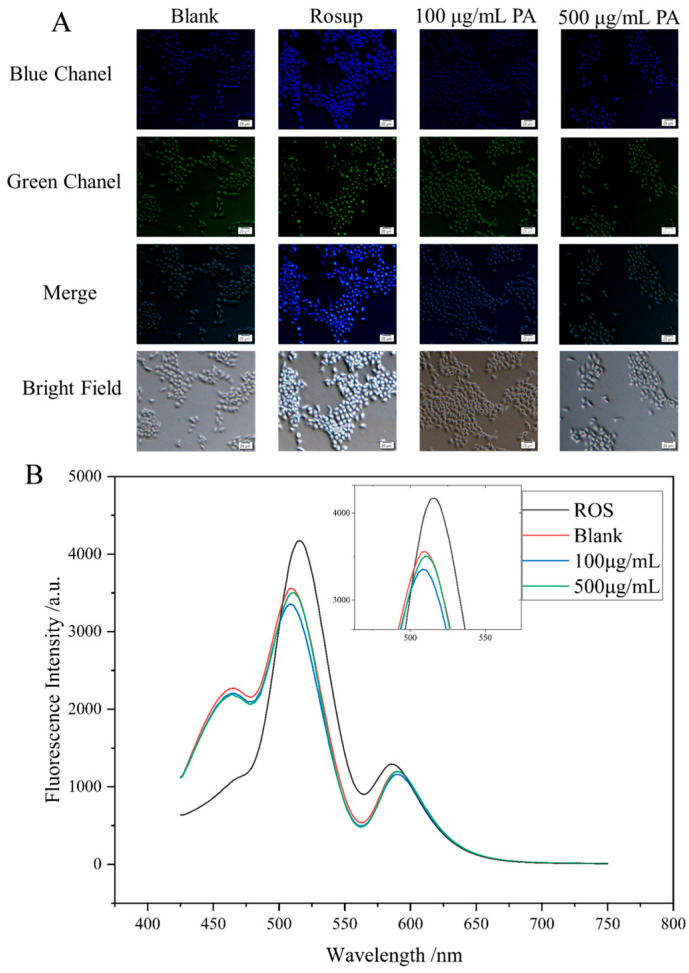
Detection of intracellular ROS levels in HaCaT cells using the redox-sensitive dye DCFH-DA. (**A**) Representative fluorescence images of HaCaT cells treated with PAE at different concentrations. Blue fluorescence (blue channel) indicates intracellular ROS levels, while green fluorescence (green channel) represents nuclei stained with a nuclear dye. Merge and bright-field images are also shown. (**B**) Fluorescence intensity spectra of DCF in HaCaT cells measured using a microplate reader. ROS levels increased in the Rosup group and decreased with PAE treatment in a dose-dependent manner.

The intracellular levels of reactive oxygen species (ROS) were quantified using the redox-sensitive dye 2′,7′-dichlorodihydrofluorescein diacetate (DCFH-DA) (Figure 7A). Once inside the cells, DCFH-DA is hydrolyzed by esterases to produce DCFH, which is then oxidized to the fluorescent compound DCF by ROS. The fluorescence intensity, which serves as an indicator of ROS levels, was measured using a fluorescence spectrometer (Figure 7B). Statistical analysis of the fluorescence intensity data indicated a significant decrease in intracellular ROS accumulation in HaCaT cells that were pretreated with Polyphenolic Acetate Extract (PAE) for six hours in comparison to the control group. These results suggest that PAE effectively mitigates oxidative stress by reducing ROS generation, reinforcing its potential cytoprotective effects. Furthermore, the enhanced cell viability noted in PAE-treated HaCaT cells reinforces the idea that PAE provides protective effects mainly by diminishing oxidative damage, thereby facilitating cellular survival in the face of oxidative stress. Together, these findings highlight the therapeutic potential of PAE in alleviating oxidative stress-related cellular damage and improving cell viability.

Utilizing advanced immunofluorescence microscopy techniques, the expression and subcellular localization of Wnt-10b and β-catenin in HaCaT cells, both pivotal regulators of the Wnt-10b signaling pathway, were examined in detail. The findings unveiled distinct expression patterns and cellular localizations for each protein, providing insights into their potential roles in cellular signaling and function.

Immunofluorescence analysis revealed that Wnt-10b is predominantly localized in the cytoplasm of HaCaT cells. This suggests that Wnt-10b may primarily function as an extracellular ligand, initiating the Wnt-10b signaling cascade through interaction with receptors at the cell membrane. Its cytoplasmic presence supports its involvement in paracrine signaling events that modulate essential cellular processes, including cell proliferation, differentiation, and migration, thereby potentially influencing skin homeostasis and repair mechanisms.

**Figure 8:**
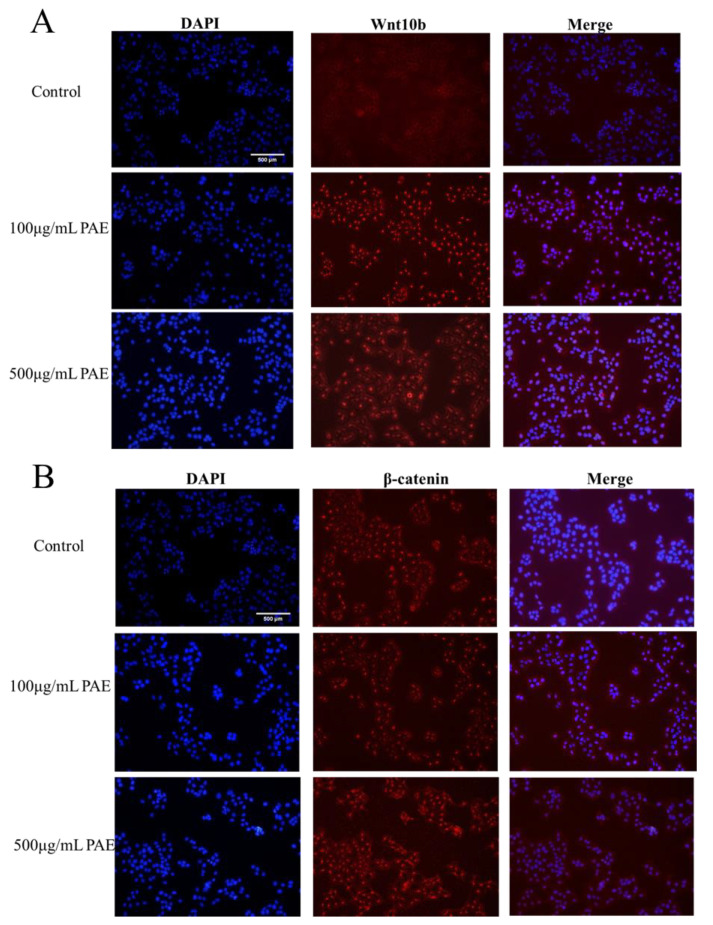
Effects of PAE on Wnt/β-catenin pathway activation. (**A**) β-catenin expression under PAE treatment. (**B**) Wnt10b expression under PAE treatment. Blue fluorescence indicates nuclei stained with DAPI, red fluorescence represents target protein (Wnt10b or β-catenin) localization, and purple in merged images shows their colocalization.

Conversely, β-catenin exhibited a dual localization pattern. In the cytoplasm, it existed within a complex of various regulatory proteins, which is consistent with its role in modulating cell-cell adhesion and mediating the canonical Wnt-10b signaling pathway. Notably, β-catenin also accumulated in the nucleus of a subset of HaCaT cells, indicative of Wnt-10b pathway activation. This nuclear translocation of β-catenin is a hallmark of the canonical Wnt-10b signaling cascade, wherein β-catenin acts as a co-activator of transcription factors to regulate gene expression critical for cell fate determination, proliferation, and differentiation. The findings suggest that Wnt-10b and β-catenin play a crucial role in regulating the behavior of HaCaT cells through both non-canonical and canonical Wnt-10b signaling pathways. The presence of Wnt-10b in the cytoplasm, along with the accumulation of β-catenin in the nucleus, underscores their functional interaction in modulating cellular responses. This study highlights the significance of immunofluorescence microscopy in elucidating the dynamic roles of signaling molecules within cellular environments. It provides valuable insights into the potential functions of Wnt-10b and β-catenin in skin biology, including processes such as wound healing, regeneration, and possibly tumorigenesis (Figure 8). Although the observed biological activities (including enhanced HaCaT proliferation and accelerated wound healing in vivo) suggest a possible involvement of the Wnt/β-catenin pathway, direct molecular validation was not performed in this study. Future work will aim to confirm this hypothesis through qPCR and Western blot analysis of key downstream targets, such as β-catenin, wnt-10b.

**Figure 9:**
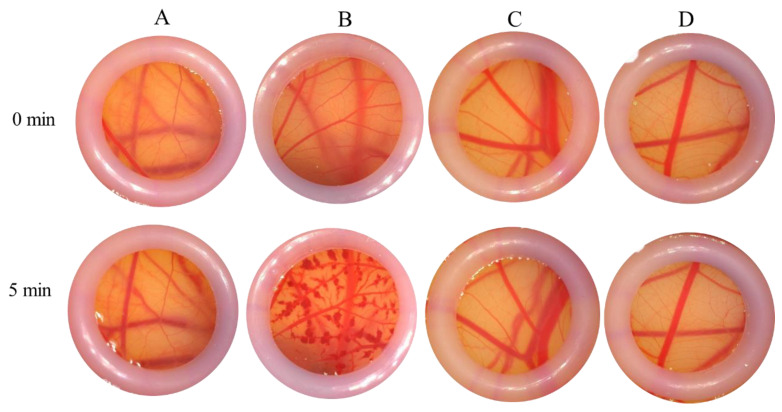
Photographs from CAM assay taken during vascular morphological observation of chick embryos: (**A**) negative control (0.9% normal saline); (**B**) positive control (4% SDS); (**C**) PAE (1 mg/mL); (**D**) PAE (10 mg/mL).

According to the endpoint scoring method utilized for ES value calculation, the stimulating conclusions for each subject were derived. The negative control exhibited no hyperemia and no exudation and maintained a clear morphological structure and profile. In contrast, the positive control demonstrated marked hyperemia, significant fading, and hemoglobin degeneration. The PAE group showed slight hyperemia with no exudation, maintaining a clear morphological structure and profile, with no significant changes observed. Morphological observations indicated that the vessel structure in the PAE groups remained clear, without hyperemia or exudation at the capillary endings. When compared to the control group, all PAE products were assessed as non-irritating or only mildly irritating (Figure 9).

**Figure 10:**
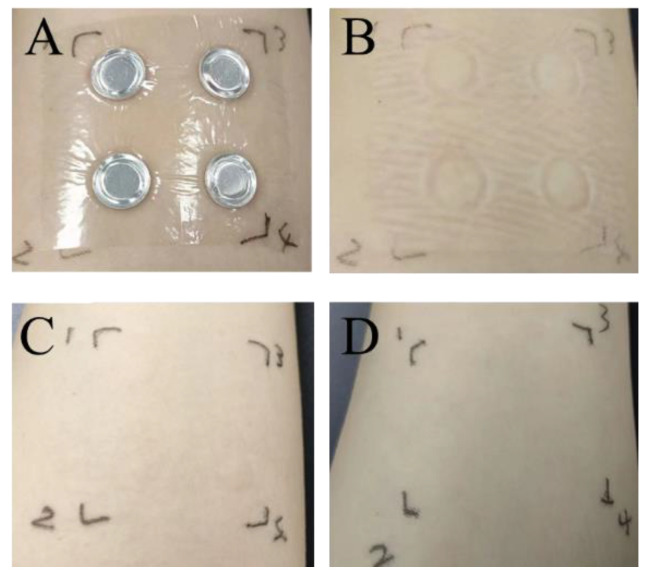
Patch application example analysis diagram (**A**) patch placement diagram; (**B**) 30 min post-patch removal; (**C**) 24 h post-patch removal; (**D**) 48 h post-patch removal.

The findings from the human skin patch test are detailed in Figure 10 and Table 1. No changes, including even mild erythema, were noted on the skin of the 30 participants, suggesting that PA extracts are safe for use on human skin. Overall, all safety evaluation results confirmed that the PAE is sufficiently safe for human skin applications. No visible reaction (score = 0) was observed in any of the 30 participants at any time point or concentration, confirming the absence of irritation and excellent skin compatibility of PAE.

**Figure 11:**
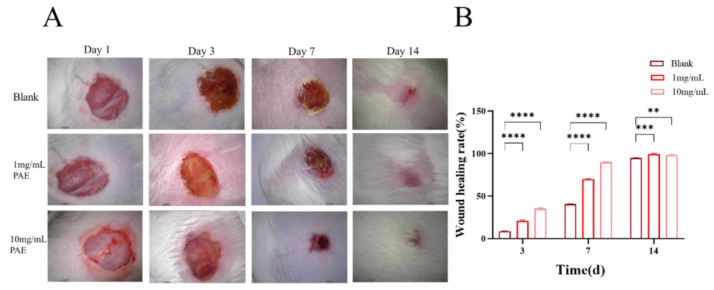
In vivo wound healing effects of *Periplaneta americana* extract (PAE) on full-thickness skin wounds in mice. (**A**) Representative wound healing photographs on days 3, 7, and 14 after treatment with normal saline (NC), 1 mg/mL PAE, and 10 mg/mL PAE. (**B**) Quantitative analysis of wound healing rates under different treatment conditions over time. ** *p* < 0.01, *** *p* < 0.001,**** *p* < 0.0001.

The excision wound model studies indicated that the control mice experienced a wound contraction rate of 8.27% from day 1 to day 3 and 40.4% from day 7 to day 14. In contrast, the PAE exhibited a steady increase in wound contraction, starting at 20.42% on day 1 and progressing to 98.96% by day 14. Mice treated topically with PAE demonstrated a wound contraction rate of 34.17% on day 3, increasing to 89.84% on day 7 and achieving complete wound contraction by day 14. Notably, complete epithelialization was observed in the PAE-treated groups on day 14. The findings from the excision wound models reveal that both groups treated with individual extracts showed significant improvements in restoring normal skin architecture, with complete epithelialization by day 14, underscoring the potent wound healing efficacy of the extract (Figure 11).

## 3. Discussion

Our findings reveal that water-extracted, low-molecular-weight peptides derived from *Periplaneta americana* display a distinctive array of biochemical and cellular activities that are highly pertinent to skin health. The prevalence of tri-, tetra-, and octapeptides (less than 1000 Da) likely accounts for their rapid tissue penetration and bioavailability. Additionally, the substantial presence of phenolics and flavonoids enhances direct reactive oxygen species (ROS) scavenging. Furthermore, the peptides demonstrate a 58% inhibition of hyaluronidase at a concentration of 5 mg/mL, confirming their ability to prevent the degradation of hyaluronic acid—a crucial mechanism for sustaining dermal hydration and elasticity. These compounds play a significant role in neutralizing free radicals, thereby reducing oxidative stress and mitigating skin damage. Although the exact degree of hydrolysis (DH) was not quantified in this study, BCA assay-based comparison and peptide enrichment via ultrafiltration confirmed successful proteolysis. Future work will incorporate DH quantification and correlation with biological activity.

The polyphenols and flavonoids present in PAE are believed to possess the ability to inhibit cancer cell proliferation, suggesting potential therapeutic applications in oncology. In HaCaT keratinocytes, we observed a 62.6% increase in proliferation and a 54% acceleration in wound closure, which correlate with the upregulation of Wnt-10b/β-catenin signaling—a key pathway for epidermal regeneration and barrier repair. Concurrently, a reduction in intracellular ROS indicates their dual role in antioxidative processes and mitogenic activity. In RAW 264.7 cells, the significant suppression of pro-inflammatory cytokines (TNF-α, IL-6, IL-1β) and the restoration of superoxide dismutase (SOD) and catalase (CAT) activities suggest that these peptides not only inhibit inflammatory signaling pathways but also restore endogenous antioxidant defenses, reducing the interplay between oxidative stress and inflammation. Moreover, due to their antioxidant properties, PAE exhibits notable anti-inflammatory and skin-repairing effects. The achievement of complete (100%) wound closure in vivo by day 14 exceeds the performance of many single-function agents, underscoring the clinical significance of the multifunctional profile. The absence of irritation or toxicity observed in CAM and human patch tests further validates the safety of topical application. In comparison to other insect-derived peptides and plant extracts, *Periplaneta americana* peptides provide a well-rounded array of benefits—including antioxidative, anti-inflammatory, proliferative, and matrix-protective properties—within a single formulation. By enhancing these essential skin characteristics, PAE fosters a youthful and vibrant complexion, which is highly sought after in the cosmetics industry.

Future research should focus on elucidating the structure-activity relationships of the predominant peptide motifs, optimizing formulations for improved stability and skin delivery, and assessing the long-term effects on dermal remodeling and photoprotection. As this is a preliminary study, future work should focus on peptide formulation, long-term storage stability, and delivery system development to support commercial applications. Overall, our findings position low-molecular-weight peptides derived from *Periplaneta americana* as promising multifunctional bioactives for the advancement of next-generation cosmetic and dermatological products.

## 4. Materials and Methods

### 4.1. Subsection

Monosaccharides (glucose, mannose, galactose, fucose, xylose, rhamnose, arabinose, glucuronic acid, galacturonic acid), dextrans, K_2_SO_4_, brilliant blue G, bovine serum albumin (BSA), and polyhexamethyleneguanidine hydrochloride (PHMG) were purchased from Sigma-Aldrich (St. Louis, MO, USA). Mannuronic acid, guluronic acid were obtained from Solarbio Technology (Beijing, China). The BCA Protein Assay Kit and trypsin (0.25% Trypsin-EDTA solution) were sourced from Thermo Fisher Scientific (Waltham, MA, USA) and Jiangsu Bomeda Life Science Co., Ltd. (Suzhou, China), respectively. Phosphate-buffered saline (PBS) powder, catalase (CAT), malondialdehyde (MDA), superoxide dismutase (SOD), and ELISA kits for cytokine quantification were procured from Wuhan Servicebio Technology (Wuhan, China). Commercial enzyme-linked immunosorbent assay (ELISA) kits for interleukin-6 (IL-6), tumor necrosis factor-alpha (TNF-α), and interleukin-1 beta (IL-1β) were supplied by Sola Biotechnology (Beijing, China). Enzymes including Trypsin (Jiangsu Bomeda Life Science Co., Ltd. (Suzhou, China). All solvents and chemicals were of analytical-grade purity. The BCA assay showed an increase in absorbance following trypsin hydrolysis. This is likely due to the enhanced exposure of amino acid residues and peptide bonds, which react more readily with the BCA reagent, and is considered an indirect indication of effective proteolysis.

### 4.2. Preparation of Peptides from Periplaneta americana Aqueous Extract

One gram of *Periplaneta americana* powder was dispersed in 20 mL of distilled water and incubated in a 45 °C water bath with continuous stirring at 200 rpm for 1 h. Subsequently, 2000 U of trypsin was added, and enzymatic hydrolysis was performed at 37 °C and pH 7.0 for 2 h, with the pH adjusted hourly. The reaction was terminated by heating the mixture to 65 °C.The enzyme-to-substrate (E/S) ratio was expressed on a weight-to-weight (*w*/*w*) basis. Trypsin was added at 1% (*w*/*w*) of the *P. americana* powder, corresponding to an E/S ratio of 1:100 (*w*/*w*). The hydrolysate was then ultrasonicated at 40 °C for 70 min to extract active components effectively and subsequently centrifuged at 3000 rpm for 10 min to obtain the supernatant. Lipids were eliminated by mixing the supernatant with petroleum ether in a 1:1 volume ratio under sealed conditions for 24 h. After solvent recovery, the aqueous phase was concentrated through rotary evaporation at 45 °C to form a viscous extract. This concentrate was pre-frozen at −75 °C for 24 h, lyophilized for 48 h, and then ground into a fine powder. A 1 mg/mL peptide solution was prepared by dissolving the powder in distilled deionized water (dd H_2_O). For molecular weight fractionation, 300 μL of this solution was ultrafiltered (3 kDa cutoff) via centrifugation at 12,000 rpm and 4 °C for 10 min, with the filtrate (<3 kDa peptides) collected. The peptide concentration was quantified using a Pierce™ Quantitative Colorimetric Peptide Assay Kit (Appleton, WI, USA); standards and samples (20 μL each) were mixed with 180 μL of the working reagent in a microplate, homogenized for 30 s using an orbital shaker, incubated at 37 °C for 15 min, and then analyzed at 480 nm using a microplate reader. For reduction and alkylation, 20 μg of peptide was diluted to 100 μL with dd H_2_O, reduced with 10 mmol/L DTT at 37 °C for 1 h, and then alkylated with 50 mmol/L IAA (protected from light) for 30 min. The mixture was desalted using self-packed columns and dried under vacuum centrifugation at 45 °C.

### 4.3. Characterization of PAE

#### 4.3.1. LC-MS Identification of Monosaccharide Determination

Standard solutions containing known concentrations of monosaccharides were prepared to construct calibration curves for quantitative analysis, adhering to established protocols [18,19]. For sample preparation, 100 mg of lyophilized peptide powder from the aqueous extract of *Periplaneta americana* was accurately weighed into a 2.0 mL microcentrifuge tube, mixed with 700 μL of 80% ethanol, and incubated at 50 °C with shaking for 2 h. Following incubation, an additional 700 μL of deionized water was added to dilute the extract, after which the mixture was centrifuged at 10,000 rpm for 3 min. The resulting supernatant was carefully transferred to a new centrifuge tube for subsequent analysis. This supernatant was initially subjected to ion chromatography for a preliminary assessment. Based on the chromatographic profile obtained, the necessary dilutions were prepared and re-analyzed to ensure accurate quantification. Monosaccharide analysis was conducted using a Thermo Scientific ICS-5000+ ion chromatography system (Thermo Fisher Scientific, USA) equipped with a Dionex™ CarboPac™ (*Waltham, MA, USA*) PA10 analytical column (250 mm × 4.0 mm, 10 µm). The injection volume was set to 5 µL. The mobile phase was composed of solvent A (deionized water) and solvent B (100 mM sodium hydroxide), with the column temperature maintained at 30 °C. Detection was performed using an integrated electrochemical detector. Calibration curves generated from the monosaccharide standards facilitated both qualitative identification and quantitative determination of monosaccharide components in the *Periplaneta americana* extract. Monosaccharide concentrations were calculated by comparing the peak areas of the sample chromatograms with those of the corresponding standards.

#### 4.3.2. LC-MS/MS Identification of Polypeptide Components

A 200 µL aliquot of SDT lysis buffer was added to the sample, followed by sonication for 5 min (30 W, with intervals of 5 s on and 5 s off). The sample was then placed in a boiling water bath for 5 min and centrifuged at 14,000× *g* for 15 min to collect the protein supernatant.

The liquid chromatography column (0.15 mm × 150 mm, RP-C18, Column Technology Inc., Lombard, IL, USA) was equilibrated with a 95% solution A mixture. The sample was loaded onto Zorbax 300SB-C18 peptide traps (Agilent Technologies, Wilmington, DE, USA) using an autosampler and then separated on the liquid chromatography column. The enzymatic digestion products were subsequently separated by capillary high-performance liquid chromatography (HPLC) and analyzed by mass spectrometry with a Q Exactive HF-X mass spectrometer (Thermo Fisher).

#### 4.3.3. Quantitative Profiling of Primary Bioactive Constituents

The neutral carbohydrate content in the extract of *Periplaneta americana* (PAE) was quantified using the phenol-sulfuric acid method [20], with D-glucose as the reference standard. Absorbance measurements were taken at 490 nm using a microplate reader, allowing for the carbohydrate concentration to be extrapolated from a six-point calibration curve. The protein content was determined spectrophotometrically at 562 nm using a BCA assay kit, with bovine serum albumin (BSA) serving as the standard. Each sample was measured in triplicate, and total protein levels were expressed as milligrams of BSA equivalents per gram of lyophilized PAE (mg BSA eq/g).

#### 4.3.4. Total Phenolic Contents

Standard volumes of gallic acid were dissolved in 0.1 g of ethanol, and the mixture was thoroughly combined with the Folin-Ciocalteu reagent [21,22,23]. After a 5-min reaction period, 2.5 mL of a 5% sodium carbonate solution was added to terminate the reaction. The solution was mixed thoroughly and allowed to react for an adequate duration to ensure complete chromogenic development. The absorbance of the resulting solution was measured using a UV-Vis spectrophotometer at approximately 760 nm. The total polyphenol content was determined using a gallic acid calibration curve, with results expressed as the polyphenolic equivalent of the quality fraction.

#### 4.3.5. Total Flavonoid Contents

In brief, a calibration curve of rutin was plotted [24,25]. A total of 1 mL of the PAE was mixed with 5 mL of 2% AlCl_3_ in methanol. After a 60-min incubation at 23 ± 2 °C, the absorbance was determined at 490 nm. The total flavonoid content was calculated using the rutin calibration curve. Results are expressed as flavonoid equivalents. The results were expressed as the rutin equivalent per gram of dry weight.

### 4.4. Antioxidant Activity

#### Hyaluronidase Inhibitory Activity

The hyaluronidase inhibitory activity was evaluated using a spectrophotometric quantification method adapted from literature [26], which measures β-N-acetylglucosamine release via enzymatic degradation of sodium hyaluronate. Enzymatic assays were performed as follows: Bovine testicular Hyaluronidase (6000 U/mL in 0.1 M acetate buffer, pH 5.6) was pre-activated with 0.25 mM CaCl_2_ (100 μL) at 37 °C for 20 min. Test samples (500 μL, serially diluted in buffer) were then introduced, followed by a 20 min equilibration period. The reaction was initiated by adding 500 μL sodium hyaluronate substrate (0.5 mg/mL in acetate buffer) and maintained at 37 °C for 30 min. Reaction termination was achieved through alkaline denaturation using 100 μL 0.4 M NaOH, with immediate cooling in an ice-water bath (5 min). Chromophore development involved sequential addition of 500 μL acetylacetone reagent (3.5% *v*/*v* in 1 M Na_2_CO_3_) and thermal derivatization (100 °C, 30 min). Following equilibration to 25 °C, 1.0 mL Ehrlich’s reagent was incorporated, with 20 min ambient incubation for chromogenic stabilization. The optical density of the reaction mixture was measured at 555 nm by using a microplate reader. Inhibitory activity (%) was calculated as:

(1) $$\begin{array}{c} \mathrm{Inhibition}\;\left(\%\right)=\left[1-\left(\frac{A_{sample}-A_{blank}}{A_{control}-A_{blank}}\right)\right]\times 100 \end{array}$$

where A_sample_ was the absorbance of PA with different concentrations of PAE, and A_control_ and A_blank_ were the absorbance of the control and blank groups, respectively.

### 4.5. In Vitro and In Vivo Assessments

#### 4.5.1. Cell Viability Assay

An in vitro cell viability test was performed using a CCK-8 [27] according to the manufacturer’s instructions. Briefly, HaCaT cell suspension with a concentration of 1 × 10^4^ cells/mL was inoculated in each well of the 96-well plate at 37 °C in 5% CO_2_ for 12 h. Then, the culture medium was replaced with PAE medium and cultured for 12 h, 24 h, and 48 h. Normal cells were used as controls. At the corresponding time point, 10 μL CCK-8 solution was added to each well and incubated in the cell culture box for 1 h. Finally, the absorbance of samples containing different concentrations of PAE at 450 nm was measured with a microplate reader. The cell activity was calculated by the following equation.

(2) $$\begin{array}{c} \mathrm{Cell}\;\mathrm{activity}\;\left(\%\right)=\frac{\frac{A_{1}}{A_{blank}}}{\frac{A_{control}}{A_{blank}}}\times 100 \end{array}$$

where A_1_ was the absorbance of PA with different concentrations of PAE, and A_control_ and A_blank_ were the absorbances of the control and blank groups, respectively.

#### 4.5.2. Cell Migration Scratch Assay

The effect of PAE on the migration of HaCaT cells was evaluated by scratch assay [28]. HaCaT were seeded in 6-well plates at a density of 1 × 10^5^ cells per well, afterward, the cell was scratched with 200 μL sterile pipet tips in the middle of the well. Cell debris was washed with PBS, and then the leaching solutions were added to the cells. The images were taken under an inverted microscope after 0, 24, or 48 h of incubation. Scratched areas were analyzed with ImageJ (Image analysis was performed using ImageJ software (version 1.52a, National Institutes of Health, Bethesda, MD, USA), available at http://imagej.nih.gov/ij) and the results were calculated as the difference between scratch areas at 0 h at different times.

(3) $$\begin{array}{c} \mathrm{Scratch}\;\mathrm{healing}\;\mathrm{rate}=(W_{0}-W_{t})/W_{0} \end{array}$$

where, W_0_ represents the scratch area of 0 h, W_t_ is the scratch test to investigate the effect of PA extract on HaCaT cell migration.

#### 4.5.3. Quantitative Analysis of Cell Proliferation Using EDU Assay

HaCaT Cells were cultured in DMEM supplemented with 10% FBS and 1% penicillin-streptomycin. Cells were maintained at 37 °C in a humidified atmosphere containing 5% CO_2_ until they reached 70–80% confluence. Cells were seeded into 6-well plates at a density of 1 × 10^5^ cells per well and incubated for 24 h to allow for attachment. After 24 h, cells were treated with EDU solution at a final concentration of 10 µM for 2 h at 37 °C to label the newly synthesized DNA. Following EDU labeling, cells were fixed with 4% paraformaldehyde in PBS for 15 min at room temperature. Fixed cells were then washed three times with PBS and permeabilized with 0.5% Triton X-100 in PBS for 20 min. Cells were subjected to a click reaction using a commercially available Click-iT EDU imaging kit following the manufacturer’s protocol. This involved incubating cells with a Click-iT reaction cocktail containing copper sulfate, fluorescent azide, and ascorbic acid for 30 min at room temperature, protecting from light. After the click reaction, cells were washed with PBS and incubated with DAPI solution for 5 min at room temperature to counterstain the nuclei. Cells were then washed three times with PBS to remove excess DAPI. Stained cells were visualized using a fluorescence microscope. Images were captured, and EDU-positive cells (indicating proliferating cells) were counted in at least five random fields per well. The percentage of EDU-positive cells was calculated by dividing the number of EDU-positive cells by the total number of DAPI-stained nuclei and multiplying by 100. Results were expressed as mean ± SD from three independent experiments.

### 4.6. Detection and Localization of Wnt/β-Catenin Signaling Pathway Expression by Immunofluorescence in Cells

HaCaT cells were cultured in DMEM supplemented with 10% FBS and 1% penicillin-streptomycin. The cells were maintained at 37 °C in a humidified incubator with 5% CO_2_ until they reached 70–80% confluence. For immunofluorescence analysis, cells were washed with PBS and fixed in 4% paraformaldehyde for 15 min at room temperature. After fixation, cells were washed three times with PBS and permeabilized with 0.1% Triton X-100 for 10 min. To block non-specific antibody binding, cells were incubated with 5% BSA in PBS for 1 h at room temperature. Cells were then incubated overnight at 4 °C with primary antibodies targeting β-catenin and other relevant Wnt signaling pathway proteins. After three washes with PBS, cells were incubated with fluorescently conjugated secondary antibodies for 1 h at room temperature, in the dark. To visualize the nuclei, cells were stained with DAPI solution for 5 min at room temperature. Following DAPI staining, cells were washed three times with PBS to remove excess stains. A drop of anti-fade mounting medium was added to the slides, and a coverslip was carefully placed on top. The slides were sealed with nail polish to prevent drying and shifting of the coverslip. Fluorescent images were captured using a confocal microscope, and the localization and expression of β-catenin were analyzed by examining the fluorescence signals.

Gene name Sequence:

Wnt 10b Forward 5′-CAAACCACTGGAGGTCCTGA-3′
Reverse 5′-CTCCTCCAGCATGTCGAAGC-3′
β-catenin Forward 5′-CAAACCACTGGAGGTCCTGA-3′
Reverse 5′-CTCCTCCAGCATGTCGAAGC-3′
GAPDH Forward 5′-AATGGGCAGCCGTTAGGAAA-3′
Reverse 5′-GCCCAATACGACCAAATCAGAG-3′

### 4.7. Reactive Oxygen Species and Inflammatory Responses

#### 4.7.1. Reactive Oxygen Species Generation and Measured in HaCaT Cells

HaCaT cells were treated with PAE at concentrations of 0.1, 1, and 10 mg/mL, with or without the addition of 0.1% Rosup. Then, the intracellular ROS levels in each group were quantified using 2′,7′-dichlorodihydrofluorescein diacetate (DCFH-DA, Beyotime) [29,30]. Cells were washed with DMEM and subsequently incubated in DMEM supplemented with 10 µM DCFH-DA at 37 °C for 20 min. Fluorescence intensity was measured using a fluorescence microscope, with the ISO set to 800 and an exposure time of 500 ms. ROS generation in HaCaT cells was quantitatively assessed using H2DCFH-DA, and fluorescence intensity was recorded using a fluorescence spectrophotometer (excitation wavelength: 488 nm, emission wavelength: 525 nm). Results were expressed as fluorescence intensity. Cell images were captured using an Olympus fluorescence inverted microscope, and high-resolution images were obtained using an Olympus fluorescence microscope (Olympus Corporation, Tokyo, Japan).

#### 4.7.2. Anti-Inflammatory Factors

The blank, control, and experimental groups were set, and the RAW264.7 cells at a density of 5.0 × 10^4^ per well were seeded in each well of the 12-well plate. At 1 mL per well, At the time of 37 °C. After 24 h of incubation in a 5% CO_2_ incubator, 1 mL of culture medium was added to the blank group. Then, another 1 mL of medium with 1 μg/mL of LPS was added to each well of the control group. Different concentrations of PA solutions were added to the experimental group. The final concentration was 1 mg/mL, 10 mg/mL, at 37 °C, 5% CO_2_. After incubation for 8 h in the incubator, the cell supernatants were collected. Determine the relevant indicators of inflammatory mediators in cells (TNF-α, IL-6, IL-1β) [31,32,33]; Tests of TNF-α by ELISA, Level of IL-6, IL-1β secretion [34,35]. The experimental data were expressed as mean soil standard deviation (mean ± SD, n = 3) and significantly analyzed by one-way analysis of variance using SPSS19.0 software.

#### 4.7.3. Quantitative Analysis of LDH, CAT, MAD, and SOD Enzyme Activities for Validation of Anti-Inflammatory Effects

HaCaT keratinocytes were cultured in Dulbecco’s Modified Eagle Medium (DMEM) enriched with 10% fetal bovine serum (FBS) and 1% penicillin-streptomycin. The cells were kept at 37 °C in a humidified incubator supplemented with 5% CO_2_. To induce inflammation, the cells were treated with lipopolysaccharide (LPS) at a concentration of 1 μg/mL for 2 h. Following LPS stimulation, the cells were exposed to varying concentrations of the test extract for an additional 24 h, with recombinant collagen serving as a positive control to validate the experimental conditions.

Catalase (CAT) activity was measured using a catalase assay kit, monitoring the decomposition of hydrogen peroxide by assessing the decrease in absorbance at 240 nm. Malondialdehyde (MDA) levels, which indicate lipid peroxidation, were quantified using a thiobarbituric acid-reactive substances (TBARS) assay, where absorbance was measured at 532 nm. The activity of superoxide dismutase (SOD) was evaluated using an SOD assay kit, with inhibition of nitroblue tetrazolium (NBT) reduction assessed at 560 nm using a multifunctional microplate reader from Molecular Device.

### 4.8. Safety Determination

Chicken chorioallantoic membrane assays and human skin patch tests [36]; were employed to assess the safety of the PA extracts. The animal procedures were conducted in accordance with the Guide for the Care and Use of Laboratory Animals (NIH publication No. 85e23, revised 1996). They received approval from the Experimental Animal Care and Use Ethics Committee of Shandong University of Traditional Chinese Medicine and Qilu University of Technology (Shandong Academy of Sciences) (approval number SWS20221109). All animals were provided with standard laboratory diets consistent with the Code of Practice for the Housing and Care of Animals Used in Scientific Procedures. This ensured that all research adhered to the widely accepted “3Rs” principles.

#### 4.8.1. Chicken Chorioallantoic Membrane (CAM) Assay

Irritation potential caused by PA extracts was assessed using the chicken chorioallantoic membrane (CAM) assay. Positive controls included 0.4% and 4% SDS, while 0.9% normal saline served as the negative control. Experimental groups comprised PAE concentrations of 100 μg/mL, 1 mg/mL, and 10 mg/mL. Observations and recordings focused on morphological changes in capillary endings, capillary networks, and vascular structures, as well as protein levels and the survival rate of the CAM. Data were analyzed utilizing SPSS version 19.0. Potential irritation by PA extracts was detected using the chicken chorioallantoic membrane assay, using 0.4% and 4% SDS as positive controls and 0.9% normal saline as the negative control. The PAE concentrations of 100 μg/mL, 1 mg/mL, and 10 mg/mL were used as the experimental groups. Morphological changes of capillary ending, capillary network, vascular, CAM, protein, and the survival rate of CAM were observed and recorded. Data were subjected to analysis using the software SPSS 19.0.

#### 4.8.2. Human Skin Patch Test

The skin toxicity of PA extracts was evaluated using the human skin patch test, following established methods. Thirty volunteers—comprising 15 females and 15 males aged between 22 and 32—were selected for the study. The extracts were applied to their arms for 24 h. Results were categorized into five grades: Grade 0 (negative reaction), Grade 1 (suspicious reaction with slight erythema), Grade 2 (slight positive reaction with erythema), Grade 3 (positive reaction with herpes), and Grade 4 (serious positive reaction with confluent herpes), in accordance with the guidelines set forth in the Hygienic Standard for Cosmetics. A sample is deemed to have caused adverse reactions if the number of Grade 1 responses exceeds five, if Grade 2 responses exceed two, or if the number of Grade 3 responses or higher is greater than one. In all other cases, the sample can be considered safe for human use.

### 4.9. Excision Wound Model

In the excision wound model, mice were anesthetized with a combination of ketamine (10%, 80 mg/kg) and xylazine (2%, 10 mg/kg) under sterile conditions. A full-thickness circular wound with a diameter of 6.5 mm was created on the shaved dorsal skin. Wound healing was assessed at 0, 3, 7, and 14 days post-injury by capturing digital images of the wound area, which were subsequently analyzed using specialized image analysis software. The percentage of wound contraction was calculated using the following equation:

(4) $$\;\begin{array}{c} \mathrm{Wound}\;\mathrm{contraction}\left(\%\right)=\left(S_{0}-S_{n}\right)\times 100 \end{array}$$

where S_0_ and S_n_ are wound areas on day 0 and the certain day; here, n = 3, 7, and 14. The epithelialization period was recorded as the time required for the dead tissue to fall off without any residual raw wound.

### 4.10. Statistical Analysis

The data are displayed as mean values accompanied by standard deviations. Statistical analysis was performed using Prism 8. To evaluate the statistical differences between groups, a one-way analysis of variance (ANOVA) was conducted in accordance with the experimental protocol, followed by Tukey’s multiple-comparison test. A *p*-value of less than 0.05 was considered statistically significant for all analyses.

## 5. Conclusions

Based on the current findings, PAE exhibits substantial natural antioxidant, anti-inflammatory, antibacterial, and wound-healing properties, underscoring its potential as a safe and effective bioactive ingredient suitable for various skin types. These diverse attributes position PAE as a promising candidate for incorporation into next-generation cosmetic and dermatological formulations. To further enhance its application potential, future research will aim to isolate and structurally characterize the active short peptides within PAE, as well as to elucidate their underlying molecular mechanisms and signaling pathways. A comprehensive exploration of structure-activity relationships will yield critical insights into optimizing the bioefficacy of PAE-derived peptides. Collectively, this research establishes a foundation for the development of innovative natural therapeutic strategies focused on promoting skin health and regeneration, offering broad prospects in cosmetic science and aesthetic medicine.

## Figures and Tables

**Table 1:** Results of the Human Skin Patch Test. No erythema, edema, or other adverse reactions were observed in any of the 30 subjects after 24 and 48 h, indicating excellent dermal compatibility of PAE.

Group	Time (Min)	No Change (0)	Light Erythema (1)	Erythema, Infiltration, Papules (2)	Edematous Erythema or Papules (3)	Significant Erythema with Pimples or Blisters (4)
Negative control	0.5	30	0	0	0	0
	24	30	0	0	0	0
	48	30	0	0	0	0
PAE (100 μg/mL)	0.5	30	0	0	0	0
	24	30	0	0	0	0
	48	30	0	0	0	0
PAE (500 μg/mL)	0.5	30	0	0	0	0
	24	30	0	0	0	0
	48	30	0	0	0	0

## Data Availability

Data are available upon request from the corresponding author.

## Supplementary Materials

The following supporting information can be downloaded at: https://www.mdpi.com/article/10.3390/molecules30142901/s1.
